# A Novel Methodology using CT Imaging Biomarkers to Quantify Radiation Sensitivity in the Esophagus with Application to Clinical Trials

**DOI:** 10.1038/s41598-017-05003-x

**Published:** 2017-07-20

**Authors:** Joshua S. Niedzielski, Jinzhong Yang, Francesco Stingo, Zhongxing Liao, Daniel Gomez, Radhe Mohan, Mary Martel, Tina Briere, Laurence Court

**Affiliations:** 1 Department of Radiation Oncology, The University of Colorado-School of Medicine, Aurora, Colorado USA; 2 Department of Radiation Physics, The University of Texas-MD Anderson Cancer Center, Houston, Texas USA; 3 Department of Radiation Oncology, The University of Texas-MD Anderson Cancer Center, Houston, Texas USA; 4 Department of Statistics, Computer Science, Applications “G. Parenti”, University of Florence, Florence, Italy; 5 University of Texas-Houston Health Science Center, Graduate School of Biomedical Science, Houston, Texas USA

## Abstract

Personalized cancer therapy seeks to tailor treatment to an individual patient’s biology. Therefore, a means to characterize radiosensitivity is necessary. In this study, we investigated radiosensitivity in the normal esophagus using an imaging biomarker of radiation-response and esophageal toxicity, esophageal expansion, as a method to quantify radiosensitivity in 134 non-small-cell lung cancer patients, by using K-Means clustering to group patients based on esophageal radiosensitivity. Patients within the cluster of higher response and lower dose were labelled as radiosensitive. This information was used as a variable in toxicity prediction modelling (lasso logistic regression). The resultant model performance was quantified and compared to toxicity prediction modelling without utilizing radiosensitivity information. The esophageal expansion-response was highly variable between patients, even for similar radiation doses. K-Means clustering was able to identify three patient subgroups of radiosensitivity: radiosensitive, radio-normal, and radioresistant groups. Inclusion of the radiosensitive variable improved lasso logistic regression models compared to model performance without radiosensitivity information. Esophageal radiosensitivity can be quantified using esophageal expansion and K-Means clustering to improve toxicity prediction modelling. Finally, this methodology may be applied in clinical trials to validate pre-treatment biomarkers of esophageal toxicity.

## Introduction

Currently, cancer therapy is rapidly moving towards personalization of treatment for individual patients^1–3^. This is particularly true in radiation oncology as radiomics and radiogenomics are active areas of research, specifically as pre-treatment biomarkers of treatment outcomes with particular focus on tumor control^4–9^. The other aspect of radiation therapy outcomes, namely normal tissue toxicity, must also be considered in personalized cancer therapy utilizing radiation^10^. In many cases, normal tissue side effects limit the safely deliverable dose to the malignancy.

Normal tissue toxicities are of particular concern in the treatment of non-small cell lung cancer (NSCLC), were acute radiation esophagitis is a dose-limiting side effect^11–13^. Radiation esophagitis is a common toxicity that greatly reduces patient quality of life^14^. In addition to negatively affecting outcomes by limiting tumor dose, radiation esophagitis can lead to interruption of treatment which also negatively affects treatment outcomes^15^. Therefore, the normal tissue toxicity, also referred at as the response, in terms of radiation esophagitis, on an individual basis, further advances the personalization of cancer treatment, and also has the potential to considerably improve treatment outcomes and patient quality of life.

One hurdle to elucidating if a potential biomarker has utility in characterizing dose-response in terms of radiation esophagitis, is the manner in which we quantify the toxicity. Typically, pre-treatment biomarkers of toxicity are investigated in multivariate predictive models by comparing models using the pre-treatment biomarker to models not utilizing the respective biomarker in the model. These models will have an outcome, toxicity severity in our case, and biomarkers are validated if the comparison shows a large improvement in predictive performance of the model by including the pre-treatment biomarker. To date, some studies have shown certain single nucleotide polymorphisms (SNPs) as potential pre-treatment biomarkers of radiation sensitivity using the methods previously described^10, 16–21^.

Traditionally, grading criteria such as the common terminology criteria for adverse events (CTCAE) has been a typical clinical method of quantifying toxicity^22^. While this has great practical importance in terms of clinical symptom management, the use of grading criteria is suboptimal for the use of outcome assessment via predictive models, as well as for investigating pre-treatment biomarkers. This is because grading criteria assign a nominal score for toxicity severity based on the patient’s perceived symptom severity and physician chosen interventions, which are subjective in nature and are non-continuous quantifications^11^. This concern highlights the need for objective endpoint measures of toxicity severity, as well as endpoints for outcome assessment that directly relate to the individual patient’s radiation-response in the esophagus. For these reasons, the need for objective imaging biomarkers has been raised in several review articles within the realm of radiation oncology^23, 24^.

The radiation-induced swelling response in the esophagus, deemed esophageal expansion, has been previously validated as an imaging biomarker of radiation-response and toxicity^25, 26^. By utilizing a baseline CT scan (the radiation therapy planning CT), as well as a CT scan acquired towards the end of treatment, the relative amount of swelling, or the expansion, can be quantified. When combined with the radiation dose information, a precise quantification of response is obtained from the radiation dose for a particular patient. This biomarker is able to objectively quantify toxicity in the esophagus, and therefore is a suitable endpoint for the validation of pre-treatment biomarkers of esophageal radiation sensitivity.

The goals of this study were: (i) to quantify the inter-patient variability of esophageal response, also referred to as the normal tissue toxicity in this study, by utilizing esophageal expansion along with the corresponding radiation dose to quantify individual patient’s dose-response; (ii) to determine if patient subgroups of radiation sensitivity can be identified in a mathematically reproducible manner using K-Means clustering; and (iii) to determine if the patient radiation sensitivity subgroup information can be used in the predictive modelling process to improve toxicity prediction models, thereby showing feasibility for this methodology as a validation procedure for pre-treatment biomarkers of radiation sensitivity.

## Methods and Materials

### Patient Population

One hundred and thirty-four patients were identified from a prospective, randomized clinical trial for the treatment of stage III NSCLC with concurrent chemoradiation therapy (paclitaxel and carboplatin), with tumor prescription doses of 60 (n = 4), 66 (n = 28), or 74 (n = 53) Gy in 2-Gy fractions over 6–8 weeks at University of Texas-MD Anderson Cancer Center. Radiation dose was chosen as the maximum of the three prescriptions that met critical structure constraints. These constraints included: mean lung dose ≤22 Gy, lung volume receiving ≥20 Gy up to 40%, mean esophageal dose ≤45 Gy, 33% of esophageal volume ≤65 Gy, 66% of esophageal volume ≤55 Gy, maximum spinal cord dose ≤50 Gy to any 2 cm^3^ volume, and mean heart dose ≤33 Gy. The inclusion/exclusion criteria included: pathologically proven, unresected stage II–IIIB NSCLC, suitability of concurrent chemo radiation therapy for treatment, age between 18 and 85 years, informed consent obtained before enrollment; small-cell histology, prior radiotherapy to the thoracic region, pregnancy. Intensity-modulated radiation therapy (IMRT) and passive-scatter proton therapy (PSPT) was utilized in 85 and 49 of the study patients, respectively. During radiation therapy, patients had weekly 4-dimensional computed tomography (4DCT) imaging and prospective esophagitis scoring according to Common Terminology Criteria for Adverse Events version (CTCAE) 3.0. Our study was approved by the University of Texas-MD Anderson Cancer Center Institutional Review Board, including obtaining informed consent for all study patients, and was compliant with Health Insurance Portability and Accountability Act (HIPAA) regulations. A summary of study patient demographics is shown in Table 1.

**Table 1:** Demographics of study patients (n = 134).

Characteristic	Datum
Median age (range)	
All	66 (38–85)
Male	66 (43–85)
Female	65 (38–80)
Sex	
No. of Males	75
No. of Females	59
Histologic findings	
Squamous cell carcinoma	47
Adenocarcinoma	75
Large cell carcinoma	5
Other	7
Smoking history	
Current smoker	44
Former smoker	79
Never smoked	11
Stage	
IIa	5
IIb	9
IIIa	59
IIIb	56
IV	5
Treatment dose, Gy	
74	88
66	38
60	8
Maximum Esophagitis Grade	
Grade 0	33
Grade 2	75
Grade 3	26

CT scans were acquired on General Electric Lightspeed Discovery ST, Lightspeed RT16 (GE Healthcare, Waukesha, WI), or Philips Brilliance 64 (Philips Healthcare, Bothell, WA) CT scanners operated at 120 kV. Voxel dimensions were 0.98 × 0.98 × 2.50 mm in the right-left direction, anterior-posterior, and superior-inferior direction, respectively, with a 512 × 512-pixel area. Patient treatment planning and segmentation was conducted using the Pinnacle treatment planning system (Phillips Healthcare), with esophageal contours segmented from the cricoid cartilage to the gastroesophageal junction, in the axial plane, with Pinnacle version 9.8.

### Quantification of Esophageal Response

**Figure 1:**
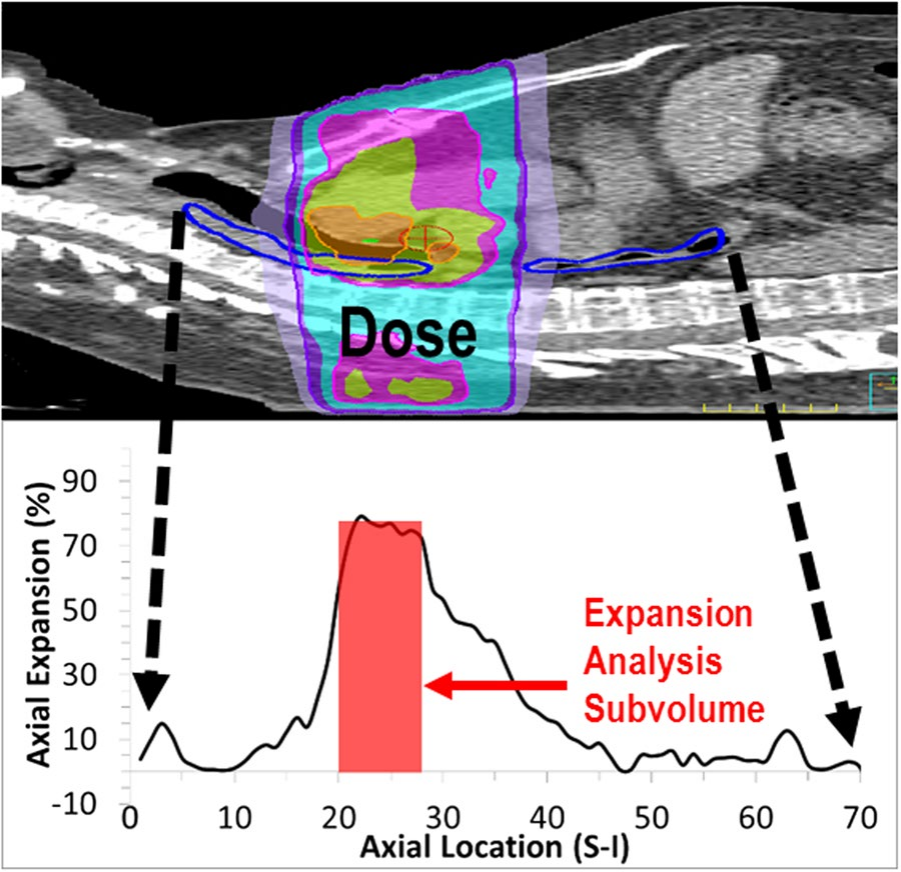
An example of esophageal expansion. (Top) Example patient thoracic CT with a colorwash of the radiation therapy dose distribution and esophageal segmentation (blue). (Bottom) Axial profile of esophageal expansion calculated at along the superior-inferior direction of the esophagus. The shaded red box is the esophageal subvolume region of analysis. Note the high expansion in the corresponding region of high radiotherapy dose.

Esophageal expansion was previously validated as a radiation-response measure in the esophagus^25^. Expansion is a surrogate quantification of esophageal swelling that is measured from relative volume change, as represented by a corresponding pair of 4DCTs (radiotherapy planning CT and CT acquired at the end of radiation therapy). An example of esophageal expansion is illustrated in Fig. 1.

The expansion-response for a given patient was quantified as the mean expansion and corresponding mean delivered radiation dose, to an isotropic esophageal sub-volume, centered at the slice location of maximum axial expansion. To maintain uniform sampling, expansion was quantified at the imaging time point nearest fraction 30 (mean = 30.5, standard deviation = ±2.2). Delivered dose was quantified as the voxel dose at the time of the expansion quantification; this is typically less than the planning dose, as fraction 30 was not the last fraction of treatment for many of the study patients. The combination of expansion value and corresponding delivered dose at the time of expansion quantification is the expansion-response for a given patient.

The underlying premise in utilizing clustering to identify patient sub-groups of differing radiosensitivity is that a particular cluster must have a proportionally higher expansion per delivered dose than other clusters. Based on the previous assumption, we assume that the 3 following clusters should be observed based on radiosensitivity: the radiosensitive cluster, which has the highest expansion per delivered dose; the radioresistant cluster, which has high delivered dose, but proportionally lower expansion than the radiosensitive group; and third, the radionormal cluster, which has lower expansion and delivered dose than the two other clusters.

The expansion dose-response quantified at the end of treatment, around fraction 30, were clustered separately using a K-Means mixture model^27^. This method is a variation of clustering using Gaussian mixture modelling, which is a process of identifying membership of the patients to a finite number of unique clusters, based on the assumption that the observed data distribution is a collection of multiple Gaussian distributions. These unique underlying Gaussian distributions are representative of the patient radiosensitivity clusters we seek to identify.

K-Means clustering is a commonly utilized technique where the squared Euclidean distance is used as a dissimilarity measure^27–29^. Minimization of dissimilarity for data points (patients) in a given number of clusters is used to find the solution. Once minimized, the patients are clustered into unique groups based on Gaussian mixture modeling of the expansion-response.

Before clustering was calculated, patients with a sub-volume dose less than 20 Gy were excluded from the analysis (n = 8). This is because there was insufficient dose to incite an expansion-response. All excluded patients were asymptomatic. After clustering the remaining 126 patients, the radiosensitive patient cluster was identified and then used in the toxicity prediction modelling process.

### Toxicity Prediction Modelling

**Figure 2:**
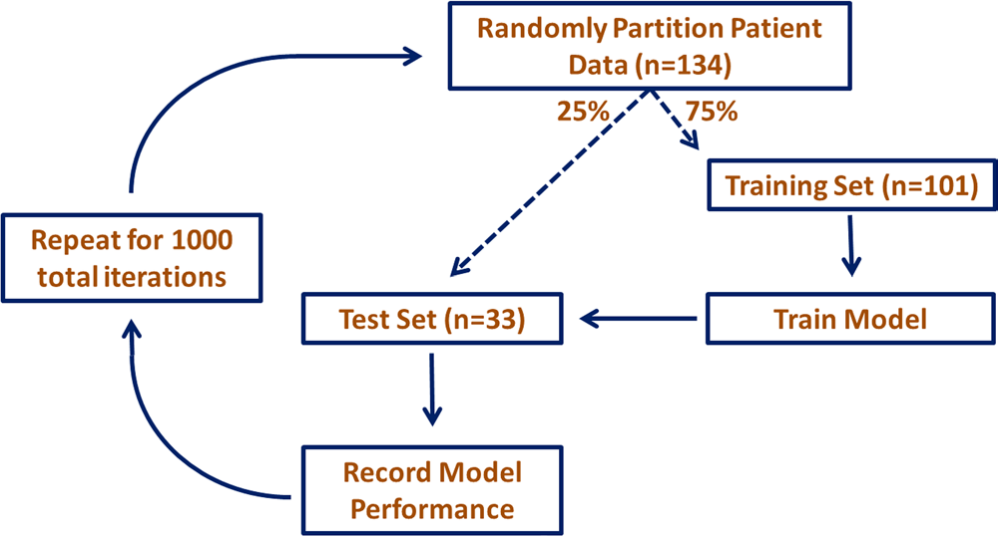
Illustration of the iterative toxicity prediction model construction process using repeated cross-validation.

In this study, least absolute shrinkage and selection operator (lasso) logistic regression was utilized to create the toxicity prediction models. These models were then used to determine if the radiosensitivity cluster membership substantially improves esophagitis prediction modelling. Lasso logistic regression is a robust model building method that prevents overfitting^28, 30^. Lasso toxicity prediction models were constructed with the 126 study patients that had adequate esophageal dose for analysis in a repeated cross-validation procedure, for 1000 iterations, which is illustrated in Fig. 2. The repeated cross-validation  procedure yields an accurate representation of model generalizability and reduces the effect of random partitioning of patient data^28, 30, 31^.

To summarize this procedure, predictor variables in the form of dosimetric and clinical factors were used as covariates to create toxicity prediction models for ≥grade 3 esophagitis complication, according to CTCAE version 3.0. Approximately 75% of the patients are randomly drawn into a training set, which builds the prediction model. The remaining 25% of patients comprise the test set and are used to quantify the built model’s predictive performance in the form of area under the curve (AUC) and Brier scores. This process is repeated for 1000 iterations to remove any influence of random draw of  patients for the training or tests sets. The dosimetric and clinical factors utilized as covariates in the models are shown in Table 2. The recurrence of model features was quantified by recording variables in each model, for every iteration of the cross-validation procedure.

**Table 2:** Predictor variables used in the NTCP model construction process.

Predictor Index	Predictor	Predictor Index	Predictor
1	Smoking Status	28	LE60_25%_
2	Induction Chemotherapy	29	LE50_25%_
3	GTV	30	LE40_25%_
4	Histology-other	31	LE30_25%_
5	Histology-Large Cell	32	LE20_25%_
6	Histology-Adenocarcinoma	33	LE10_25%_
7	Histology-Squamous Cell	34	V70
8	Nodal Involvement	35	V65
9	Stage-IV	36	V60
10	Stage-IIIB	37	V55
11	Stage-IIIA	38	V50
12	Stage-IIB	39	V45
13	Stage-IIA	40	V40
14	Tumor Location-Left Lateral	41	V35
15	Tumor Location-Right Lateral	42	V30
16	Tumor Location-Left Medial	43	V25
17	Tumor Location-Right Medial	44	V20
18	Tumor Location-Left Upper	45	V15
19	Tumor Location-Right Upper	46	V10
20	Gender	47	MED
21	Age	48	Dmax
22	LE60_100%_	49	Prescription Dose
23	LE50_100%_	50	Radiosensitivity Tag
24	LE40_100%_		
25	LE30_100%_		
26	LE20_100%_		
27	LE10_100%_		

*Abbreviations*: MED = mean esophagus dose; Dmax = maximum esophagus dose; V10 = volume of esophagus receiving at least 10 Gy; LE10_25%_ = esophageal length with at least 10 Gy to at least 25% of the cross-sectional area to axial slice of the esophagus; LE10_100%_ = esophageal length with at least 10 Gy to at least 100% of the cross-sectional area to axial slice of the esophagus.

The prediction model construction process was then repeated with radiosensitivity as an additional covariate. The previously described clustering technique was used to identify patients that had proportionally higher expansion-response then other study patients, and this information was quantified as a dichotomous variable (1 for radiosensitive patient, 0 otherwise) in the LASSO toxicity prediction modelling construction process. Model performance was assessed and recurring model predictors were cataloged for every iteration of the model construction process. The results of both model construction scenarios (with and without the radiosensitivity predictor) were compared.

### Computational Implementation

Predictor variables were standardized by subtracting the mean variable value of all patients from each individual patient value, and then dividing the result by the standard deviation^32^. All computations were conducted in MATLAB version 8.2 (Mathworks, Natick, MA). K-Means clustering was computed using MATLAB’s Statistics and Machine Learning toolbox. Lasso models were constructed using the open source glmnet package implemented in MATLAB^33^. A p-value of p < 0.05 was considered statistically significant.

## Results

### Expansion-Response and Radiosensitivity Clustering

**Figure 3:**
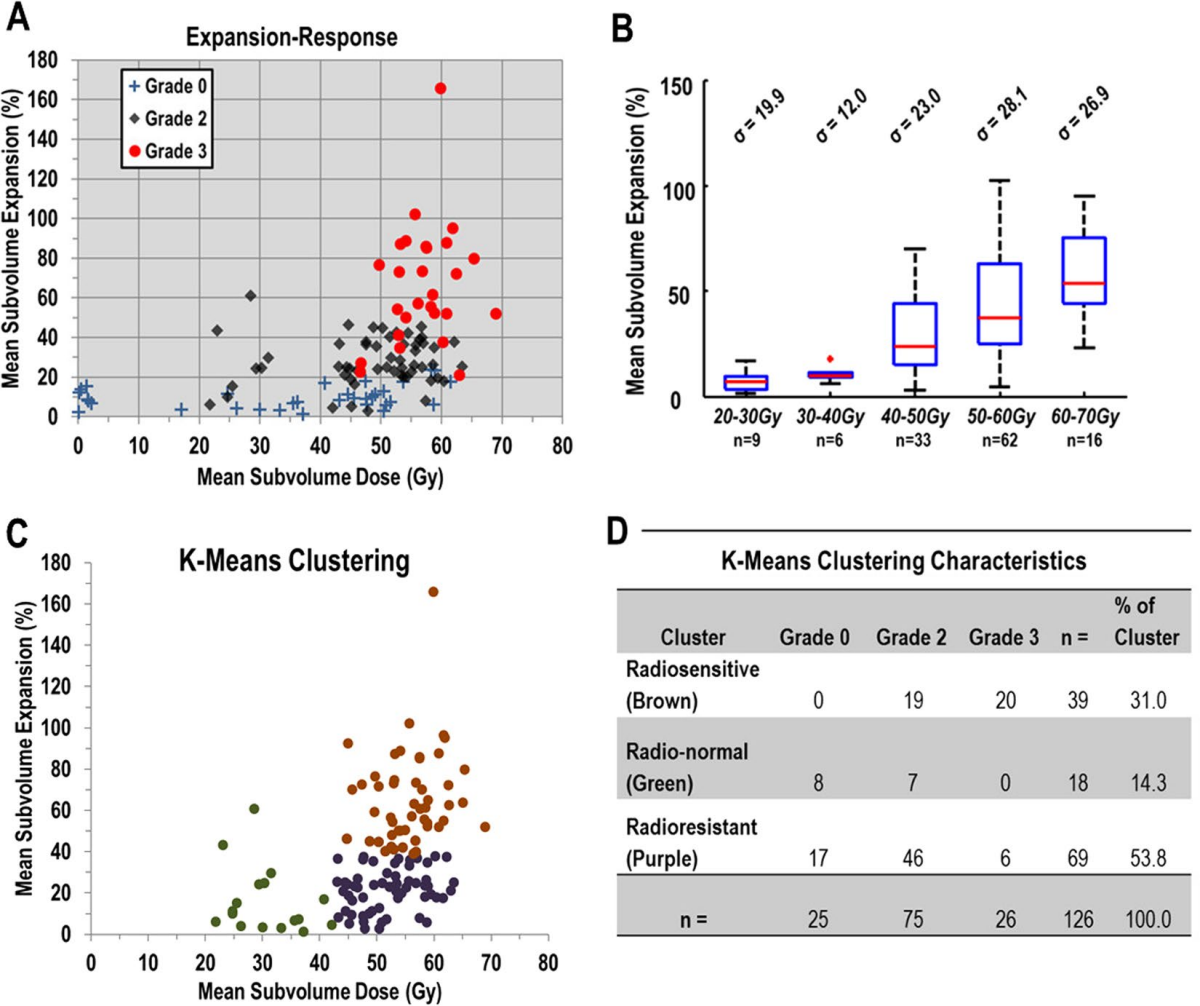
(**A**) Plot of expansion-response at the end of radiation therapy (approximately fraction 30) in the analyzed subvolume of the esophagus for 134 study patients. Patient markers denote maximum esophagitis grade during treatment. (**B**) Boxplot of the distribution of mean subvolume esophageal expansion, grouped according to mean subvolume doses of 20 to 30 Gy, 30 to 40 Gy, 50 to 60 Gy, and 60 to 70 Gy, for 126 study patients with the expansion-response quantified around treatment fraction 30. The standard deviation of expansion in each dose group is shown above each box. The edges of the box represent the quartile values of expansion, with the red line within each box representing that groups median expansion value. The range of values is represented by the black whiskers and the red ‘+’ denotes outliers (values beyond 1.5 times the interquartile range from the edge of the box). (**C**) Patient membership in radiosensitivity clusters after application of K-Means clustering on the expansion-response of the patient population. (**D**) Table of the characteristics of clustering membership for K-Means clustering for expansion-response at the end of radiation therapy for the 126 patients analyzed in the cluster analysis.

The expansion-response quantified towards the end of treatment is described for all 134 study patients in Fig. 3A. An overall trend of increasing toxicity severity along with dose is observed, but this has high patient-to-patient variability for a given dose range. Additionally, the expansion per delivered dose is also markedly variable. The distribution of expansion in 10-Gy dose partitions, from 20 Gy to 70 Gy, is shown in Fig. 3B, with a high variance of expansion observed for patients with similar doses. The standard deviation of expansion in a given dose partition is also shown, with a 30% standard deviation of expansion being typical.

The 8 patients excluded from the clustering and toxicity analyses can be identified in Fig. 3A as the patients with mean sub-volume doses under 20 Gy. The resultant K-Means clustering of the expansion-response is shown in Fig. 3C. The clusters are shown by color, with the radiosensitive cluster as brown, the radio-normal cluster is green, and the radioresistant cluster is purple. The assigned clusters’ radiation sensitivity characteristics met the necessary assumptions of expansion-response described in the methods section. Distributions of toxicity severity according to cluster membership is shown in Fig. 3D. No grade 0 patients were found in the radiosensitive cluster, but many grade 2 and 3 patients were. The radioresistant (purple) cluster contained the most patients, and all esophagitis grades being observed within this cluster.

**Figure 4:**
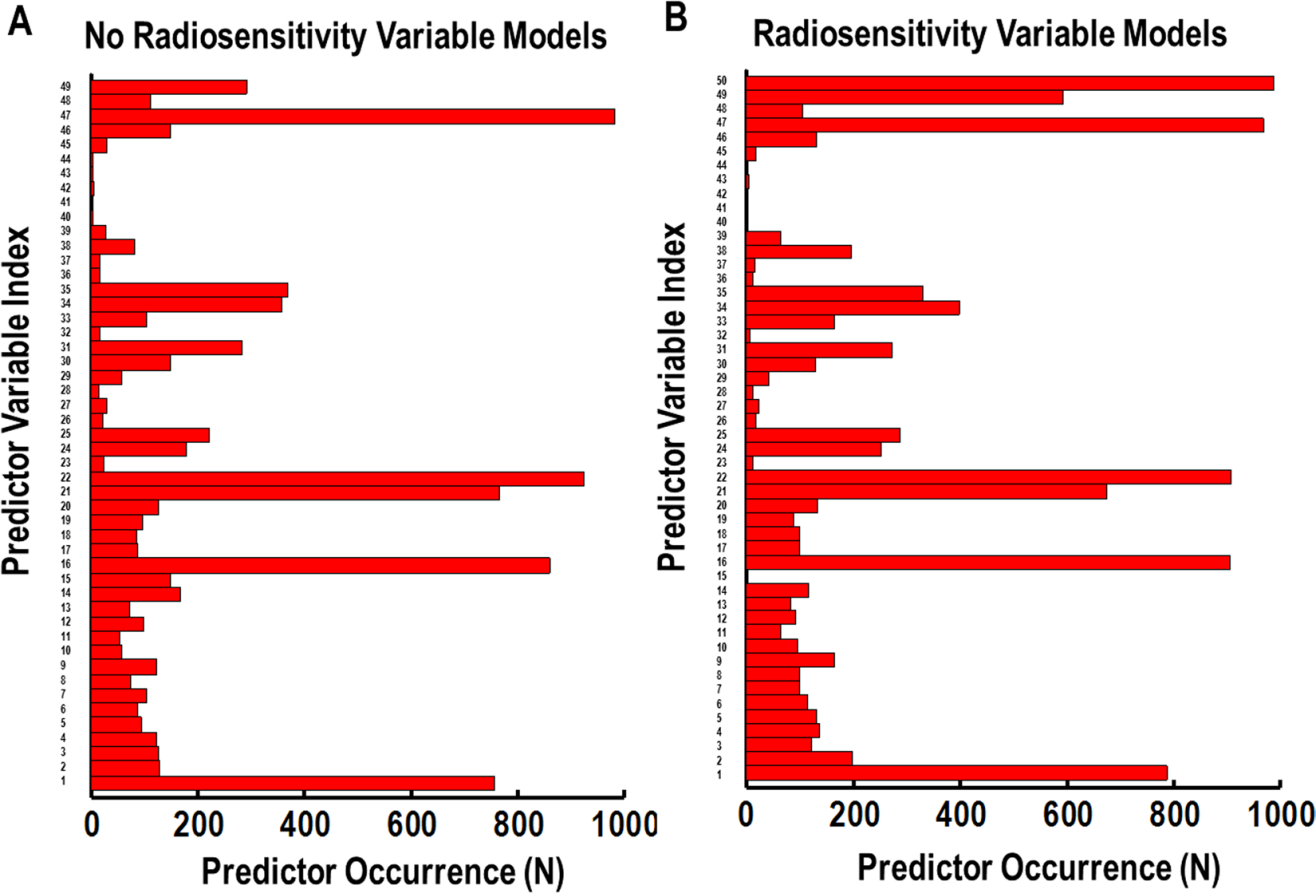
Bar charts of the occurrence of predictors for all 1000 iterations of the LASSO logistic regression toxicity prediction- modelling construction process. Models created without the radiosensitivity predictor are described in (**A**), and models using the radiosensitive tag variable from K-Means clustering of expansion-response at the end of radiotherapy are shown in (**B**). The predictor index number identifies the specific predictor variable from Table 2.

The lasso toxicity prediction model construction procedure had similar distributions of recurring model predictors, even for toxicity models not using radiosensitivity as a predictor variable. The results of predictor recurrence for models constructed without and with the radiosensitivity variable are shown in Fig. 4A and B, respectively. For models constructed with the radiosensitivity variable, this predictor was the most recurring variable and was chosen in over 99% of the 1000 iterations of model construction (top most data bar in Fig. 4B). Mean esophageal dose was the second most recurring predictor in the radiosensitivity information inclusive models and the most recurring predictor in models not including the radiosensitivity information.

The toxicity prediction model performance with and without radiosensitivity information is summarized in Table 3. Models utilizing radiation sensitivity information using K-Means clustering to identify radiosensitive patients outperformed models lacking radiosensitivity information. The training and predictive performance of models using clustering have significantly higher AUC_Training_ and AUC_Test_ (paired T-test on AUC values for each corresponding iteration of cross-validation, p < 0.05), than models not using radiation sensitivity information. Both types of Brier scores were optimal (lower value) for the clustering/radiosensitivity models compared to models without radiosensitivity information.

**Table 3:** Results of the lasso logistic regression toxicity prediction model construction process with and without using K-Means clustering to identify radiosensitive patients, from expansion-response quantified at the end of radiation therapy, for a total of 126 study patients. S.D. – Standard Deviation.

Model Type	AUC_Training_ (S.D.)	AUC_Test_ (S.D.)	Brier Score	Scaled Brier (%)
No Clustering	0.842 (±0.065)	0.693 (±0.099)	0.175 (±0.020)	18.2 (±12.0)
K-Means Clustering	0.907 (±0.055)	0.753 (±0.094)	0.151 (±0.019)	12.1 (±11.2)
**Model Type**	**Top Recurring Predictors**			
No Clustering	MED, LE50Gy_100%_, Left Medial, LE60Gy_100%_, Smoking Status			
K-Means Clustering	RS Tag, MED, LE60Gy_100%_, Smoking Status, Left Medial, Age			

The highest recurring predictors from all 1000 iterations of the model construction process are listed from highest to lowest recurring. Standard deviation of AUC values are listed in parentheses.

## Discussion

In this study, K-Means clustering was performed on esophageal expansion-response to identify patients’ inherent radiosensitivity. Clustering was calculated using expansion-response at approximately the 30th radiation therapy treatment fraction. This information was then used to identify radiosenstive patients, and this information was then converted to a dichotomous variable. This radiosensitivity information was used in the toxicity prediction modelling process in an attempt to improve esophagitis prediction models, using lasso penalized logistic regression in a repeated cross-validation procedure.

The expansion-response of these patients was highly variable regardless of delivered esophageal dose. For similar subvolume doses, many patients had vastly different amounts of expansion, in addition to varying toxicity severity. This shows a potential pitfall of toxicity prediction modelling without accounting for inherent radiation sensitivity, where variability of patients’ response outweighs the study population’s average observed response. The variability of response for patients with similar delivered dose may make detecting such effects arduous if patient radiosensitivity is not considered.

Toxicity prediction models using radiosensitivity predictor variables outperformed toxicity models not utilizing radiosensitivity information, for a grade 3 maximum esophagitis endpoint. The performance of models using radiosenstivity information is even more impressive, as a total of eight low-dose, low-response, and asymptomatic patients were excluded from the model construction process. In typical modelling scenarios, these types of patients are easily classified and will contribute to a higher model performance metric value, which would be reflected in the quantification of predictive ability. By not including these patients, the modelling situation is more challenging for the classification of esophagitis severity. Since the toxicity models with radiosensitivity information had high predictive ability despite this challenging scenario, this translated into more robust toxicity prediction models when including the radiosensitivity information.

**Figure 5:**
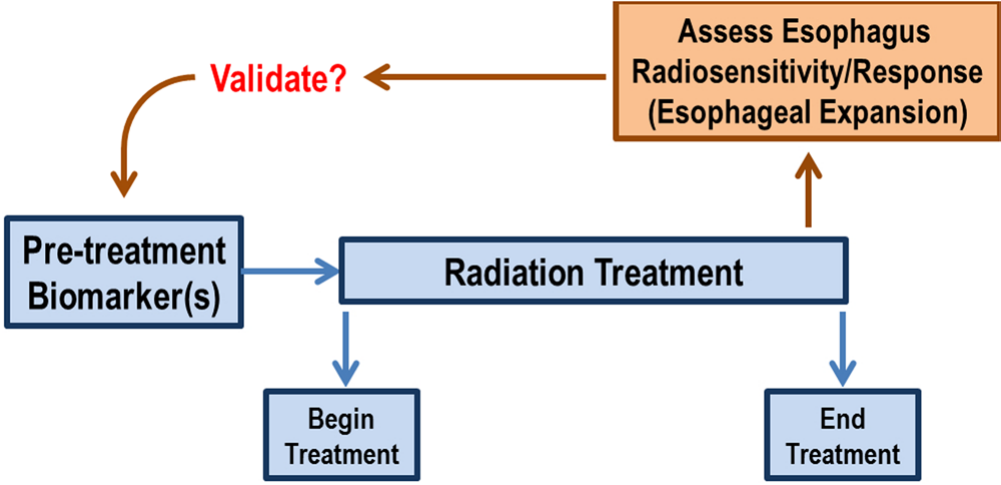
Application of esophageal expansion imaging biomarker to validate pre-treatment biomarker.

A prime application of the framework presented in this study, is the use the use of radiosenstivity, as quantified by the expansion dose-response, as a validation methodology for clinical trials. With the push into radiogenomics and a desire to quantify pre-treatment biomarkers to aid in personalized medicine, the question of how to validate such pre-treatment biomarkers arises. Traditional esophagitis grade endpoints are quite subjective and variable, and do not objectively quantify radiation-response. Since esophageal expansion is an objective radiation-response biomarker, we can utilize the expansion-response to validate any prospectively investigated pre-treatment biomarker of radiation-response, when esophageal response is a trial endpoint. A simple workflow of this concept is shown in Fig. 5. Furthermore, this validation methodology could be applied to any situation that prospectively investigates any two radiation therapy treatments (different modality, fractionation scheme, etc.) to objectively validate any difference in radiation-response in the esophagus.

This work was not without limitations. The clustering process is unsupervised in terms of esophagitis outcome, and therefore requires some assumptions for interpretation. As described in the methods, the cluster assignment of radiosensitivity was determined based on the assumptions of the relative expansion-response within the study population. It is vital to validate these findings on an external dataset, as it would be interesting to see if cluster assignment and shape would vary with new patient data. Another limitation was that the radiosensitivity information was only used dichotomously (radiosensitive or not radiosensitive). It would be of interest to analyze the utility of not just the radiosensitive clusters, but also patients labelled as radionormal and radioresistant. The radioresistant cluster in particular would be of interest in dose-escalation studies.

In addition to expansion, other types of imaging biomarkers of radiation-response can be used in a similar methodology as the work presented in this study. Esophageal uptake from ^18^F-fluorodeoxyglucose positron emission tomography (FDG-PET) has been shown to quantify radiation-response and toxicity in the esophagus^34, 35^. Therefore, it is feasible to apply esophageal FDG uptake with the clustering methodology to identify patients with inherent radiation sensitivity in the esophagus. Another application of this work is the analysis of any potential variation of radiosensitivity along the axial length of the esophagus. With a controlled cohort similarly irradiated along different axial regions of the esophagus, it may possible to determine any variability in response between specific esophageal sub-regions.

In conclusion, clustering techniques can be applied to the expansion-response mechanism to determine patient radiosensitivity. This radiosensitivity information can be used in the esophagitis prediction modelling process to improve toxicity prediction performance. Patient inherent radiosensitivity can be assessed towards the end of radiation therapy and may be applicable for outcome assessment in clinical trials that investigate response in the esophagus.

### Data Availability

The datasets generated during and/or analyzed during the current study are available from the corresponding author on reasonable request.

## References

[1] Kalia M (2013). Personalized oncology: Recent advances and future challenges. Metab. Clin. Exp..

[2] Hamburg MA, Collins FS (2010). The path to personalized medicine. N. Engl. J. Med..

[3] Meric-Bernstam F, Mills GB (2012). Overcoming implementation challenges of personalized cancer therapy. Nat. Rev. Clin. Oncol..

[4] Kerns SL (2014). Radiogenomics: the search for genetic predictors of radiotherapy response. Future Oncol..

[5] Fried DV (2014). Prognostic value and reproducibility of pretreatment CT texture features in stage III non-small cell lung cancer. Int. J. Radiat. Oncol. Biol. Phys..

[6] Lambin P (2012). Radiomics: Extracting more information from medical images using advanced feature analysis. Eur. J. Cancer.

[7] Larue RT (2016). Quantitative radiomics studies for tissue characterization: A review of technology and methodological procedures. Br. J. Radiol..

[8] Nakajo M (2017). Texture analysis of 18F-FDG PET/CT to predict tumour response and prognosis of patients with esophageal cancer treated by chemoradiotherapy. Eur. J. Nucl. Med. Mol. Imaging.

[9] Giganti F (2016). Prospective comparison of MR with diffusion-weighted imaging, endoscopic ultrasound, MDCT and positron emission tomography-CT in the pre-operative staging of oesophageal cancer: results from a pilot study. Br. J. Radiol..

[10] Kerns SL (2015). The Prediction of Radiotherapy Toxicity Using Single Nucleotide Polymorphism−Based Models: A Step Toward Prevention. Semin. Radiat. Oncol..

[11] Werner-Wasik M (2010). Radiation dose-volume effects in the esophagus. Int. J. Radiat. Oncol. Biol. Phys..

[12] Rose J (2009). Systematic review of dose-volume parameters in the prediction of esophagitis in thoracic radiotherapy. Radiother. Oncol..

[13] Kwint M (2012). Acute esophagus toxicity in lung cancer patients after intensity modulated radiation therapy and concurrent chemotherapy. Int. J. Radiat. Oncol. Biol. Phys..

[14] Bruner DW (2004). Outcomes research in cancer clinical trial cooperative groups: the RTOG model. Quality Life Res..

[15] Cox JD (1993). Interruptions of high-dose radiation therapy decrease long-term survival of favorable patients with unresectable non-small cell carcinoma of the lung: analysis of 1244 cases from 3 radiation therapy oncology group (RTOG) trials. Int. J. Radiat. Oncol. Biol. Phys..

[16] Kelsey CR (2012). A polymorphism within the promoter of the TGFβ1 gene is associated with radiation sensitivity using an objective radiologic endpoint. Int. J. Radiat. Oncol. Biol. Phys..

[17] Jin J (2015). Use a survival model to correlate single-nucleotide polymorphisms of DNA repair genes with radiation dose–response in patients with non-small cell lung cancer. Radiother. Oncol..

[18] Fernet M, Hall J (2004). Genetic biomarkers of therapeutic radiation sensitivity. DNA Repair.

[19] Rattay T, Talbot CJ (2014). Finding the Genetic Determinants of Adverse Reactions to Radiotherapy. Clin. Oncol..

[20] Lopez-Guerra JL (2012). Association between single nucleotide polymorphisms of the transforming growth factor β1 gene and the risk of severe radiation esophagitis in patients with lung cancer. Radiother. Oncol..

[21] Lopez-Guerra JL (2011). Functional promoter rs2868371 variant of HSPB1 associates with radiation-induced esophageal toxicity in patients with non-small-cell lung cancer treated with radio(chemo)therapy. Radiother. Oncol..

[22] Trotti A (2003). CTCAE v3.0: Development of a comprehensive grading system for the adverse effects of cancer treatment. Semin. Radiat. Oncol..

[23] Bentzen SM (2010). Quantitative Analyses of Normal Tissue Effects in the Clinic (QUANTEC): an introduction to the scientific issues. Int. J. Radiat. Oncol. Biol. Phys..

[24] Jeraj R (2010). Imaging for assessment of radiation-induced normal tissue effects. Int. J. Radiat. Oncol. Biol. Phys..

[25] Niedzielski JS (2016). Objectively quantifying radiation esophagitis with novel computed tomography-based metrics. Int. J. Radiat. Oncol. Biol. Phys..

[26] Court LE (2013). A technique to use CT images for *in vivo* detection and quantification of the spatial distribution of radiation-induced esophagitis. J. Appl. Clin. Med. Phys..

[27] Kanungo T (2002). An efficient K-Means clustering algorithm: analysis and implementation. IEEE Trans. Pattern Anal. Mach. Intell..

[28] Hastie, T., Tibshirani, R. & Friedman, J. *The elements of statistical learning: data mining*, *inference and prediction* (Springer, 2009).

[29] Jain AK (2010). Data clustering: 50 years beyond K-Means. Pattern Recogn. Lett..

[30] Xu CJ (2012). Impact of learning methods on the predictive power of multivariate normal tissue complication probability models. Int. J. Radiat. Oncol. Biol. Phys..

[31] Kuhn, M. & Johnson, K. *Applied Predictive Modeling* (Springer, 2013).

[32] Steyerberg, E. W. *Clinical prediction models: A practical approach to development*, *validation*, *and updating* (Springer, 2009).

[33] Friedman J, Hastie T, Tibshirani R (2010). Regularization paths for generalized linear models via coordinate descent. J. Stat. Softw..

[34] Niedzielski JS (2016). ^18^F-Fluorodeoxyglucose Positron Emission Tomography can quantify and predict esophageal injury during radiation therapy. Int. J. Radiat. Oncol. Biol. Phys..

[35] Mehmood Q (2016). Predicting Radiation Esophagitis Using 18F-FDG PET During Chemoradiotherapy for Locally Advanced Non–Small Cell Lung Cancer. J. Thorac. Oncol..

